# In Vivo Efficacy of Wound Healing under External (Bio)AgNCs Treatment: Localization Case Study in Liver and Blood Tissue

**DOI:** 10.3390/ijms24010434

**Published:** 2022-12-27

**Authors:** Viorica Railean, Magdalena Buszewska-Forajta, Agnieszka Rodzik, Adrian Gołębiowski, Paweł Pomastowski, Bogusław Buszewski

**Affiliations:** 1 Department of Infectious, Invasive Diseases and Veterinary Administration, Institute of Veterinary Medicine, Gagarina 7, 87-100 Torun, Poland; 2 Centre for Modern Interdisciplinary Technologies, Nicolaus Copernicus University, Wilenska 4, 87-100 Torun, Poland; 3 Institute of Veterinary Medicine, Faculty of Biological and Veterinary Sciences, Nicolaus Copernicus University in Toruń, 1 Lwowska St., 87-100 Torun, Poland; 4 Department of Plant Physiology, Genetics, and Biotechnology, University of Warmia and Mazury in Olsztyn, 10-229 Olsztyn, Poland; 5 Department of Environmental Chemistry and Bioanalytics, Faculty of Chemistry, Nicolaus Copernicus University, Gagarin 7, 87-100 Torun, Poland

**Keywords:** mouse model, silver nanocomposites, flow cytometry, liver, blood, accumulation

## Abstract

The present study reports on the in vivo application of (Bio)silver nanocomposite formulations (LBPC-AgNCs) on wound healing. Additionally, the present study emphasizes the limited uptake of silver by liver and blood tissues as well as the high viability of PBMCs following external LBPC-AgNCs treatment. The wound closure was monitored via stereoscopic microscope, a localization case study in liver and blood tissue was carried out by (Inductively Coupled Plasma–Mass Spectrometers (ICP/MS), and peripheral blood mononuclear cells (PMBC) viability was determined via flow cytometry technique. The silver formulation was applied externally on the site of the wound infection for a period of ten days. At the beginning of the experiment, a moderate decrease in body weight and atypical behavior was observed. However, during the last period of the experiment, no abnormal mouse behaviors were noticed. The wound-healing process took place in a gradual manner, presenting the regeneration effect at around 30% from the fourth day. From the seventh day, the wounds treated with the silver formulation showed 80% of the wound healing potential. The viability of PBMCs was found to be 97%, whereas the concentrations of silver in the liver and blood samples were determined to be 0.022 µg/g and 9.3 µg/g, respectively. Furthermore, the present report becomes a pilot study in transferring from in vitro to in vivo scale (e.g., medical field application) once LBPC-AgNCs have demonstrated a unique wound healing potential as well as a non-toxic effect on the liver and blood.

## 1. Introduction

The worldwide incidence of skin infection has increased significantly and affects almost one-third of the world’s population [1]. Approximately 8.2 million of all hospitalized patients worldwide have a chronic wound, with a resulting Medicare cost estimation of approximately $28.1 billion to $96.8 billion [2]. The etiologies of chronic wounds are some of the biggest challenges faced by modern medicine and pharmacotherapy worldwide. Surgical wounds have been reported as the most often-reported wounds, followed by diabetic foot ulcers, melanoma, psoriasis, and dermatitis [1,2,3]. The skin and its major cellular components are directly involved in the wound healing response and act as a primary barrier. Chronic wounds progress through hemostasis, inflammation, proliferation, and remodeling and are manifested by an interruption of the wound-healing process [2]. In the case of chronic wounds, the wound-healing ability is limited due to the reduced proliferation effect of the senescent cells. The problem of chronic wounds is significant, and researchers are encouraged to find a new, alternative treatment for instances when the wound-healing process is interrupted [3]. In this situation, it is necessary to change the treatment plan; for instance, by moving on from traditional antibiotic therapy. Therefore, in this context, mice are mostly used as biological models, especially in physiology and biochemistry studies. Moreover, the model (C57BL/6J mouse model) used in the present research is dedicated to the study with disturbances in the wound-healing response [4]. Currently, silver ions/AgNPs are used in medicine in many antiseptic formulations (e.g., spray, cream, ointment/unguent, hydrogel, etc.) and are widely applied in order to control eczema, psoriasis, and acne treatments [5,6,7]. These kinds of nanoparticles can migrate into the organism through different pathways such as the respiratory tract, digestive system, and parenteral routes. This has been demonstrated in in vivo studies [8,9], and these nanoparticles can therefore pose a risk to human health through their potential to reach any organ or tissue. They can also have potential molecular effects, such as a decrease in cellular viability, loss of membrane integrity, or the activation of a genetic program of controlled cell death (apoptosis) [10]. On the other hand, the toxicity of nanoparticles can also be considered to be a beneficial factor for humans in terms of their anti-tumor properties, anti-lymphoproliferative effect and lack of toxicity towards immune system cells [11]. However, the delivery and accumulation of silver nanoparticles are still incompletely understood. Therefore, researchers are encouraged to investigate the distribution of silver (Ag) in remote organs secondary to the application of nanoparticles (AgNPs).

The nanotechnology field continues to develop, and assessing nanoparticle toxicity is a very important aspect for advancing the application of nanoparticles in daily life. The effect of surface coatings on the cyto-, geno-, and phototoxicity of silver nanomaterials—synthesized by chemical methods—on human skin HaCaT keratinocytes has also been reported [12]. It was found that the citrate-coated colloidal silver nanoparticles at a 100 μg/mL level are not geno-, cyto-, or phototoxic. In turn, the citrate-coated powder form of silver nanoparticles is toxic [13]. Therefore, the coating surface of nanoparticles plays a crucial role in their cytotoxic effect. According to current knowledge, this hypothesis has been previously proven concerning nanocomposites synthesized by biological method. The (Bio)nanocomposites are naturally coated with organics coatings and demonstrate non-toxicity to the L929 cell line at 100 μg/mL [14].

The (Bio)silver nanocomposites used in the present report have been previously investigated for their antimicrobial potential and were reported as an antimicrobial agent with high potential [15]. However, our knowledge regarding the toxicity of synthesized nanoparticles and their migration and accumulation in internal organs is still incomplete. Additional investigations are required as to how the proposed biologically-synthesized silver composites, which are naturally coated with organic surfaces, can influence the control delivery of the silver. It is important to note that animals do not display chronic wounds in the way that humans do; however, in many cases, animal models can mirror the pathological conditions observed in humans.

Therefore, the present research continues this line of investigation and focuses the study on the following tasks: (i) wound healing examination; (ii) determining Ag accumulation (uptake in liver tissue and blood samples); and (iii) determining the viability of peripheral blood mononuclear cells (PBMCs) as a result of the exposure of the mouse model (C57BL/6J) to external LBPC-AgNCs formulation treatment.

## 2. Results

### 2.1. Animal Acclimatization, Body and Mouse Weights

**Figure 1:**
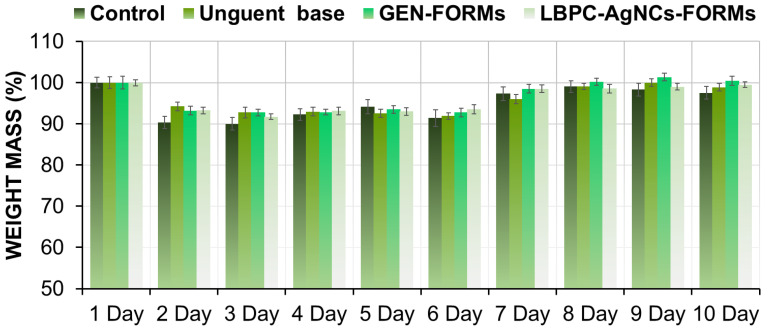
The animal weight mass represented in % during the 10 days of examination.

During the entire monitoring period of the wound-closure process, a slightly abnormal behavior with a lack of appetite was noticed during the first stage (days 1–4) of the experiment. In the middle of the experiment, starting on the fifth day, the rodents presented integration behavior and acclimatization to the environmental conditions. It is important to specify that the behavior of the mice was correlated to their body weight, manifested by a slight loss of mass (10%) at the beginning of the experiment (Figure 1).

In all cases, such observations were made until the sixth day, excepting the case in which mice were treated with the silver formulation (LBPC-AgNCs). In this case, rodents showed faster adaptation (sixth day), indicating an achievement of their initial weight compared to the control samples (untreated mice, unguent base, and GEN-FORMs) where the regeneration process started at the seventh day for GEN-FORMs and on the eighth for the rest.

### 2.2. Influence of LBPC-AgNCs Formulation on Wound Closure Rate

**Figure 2:**
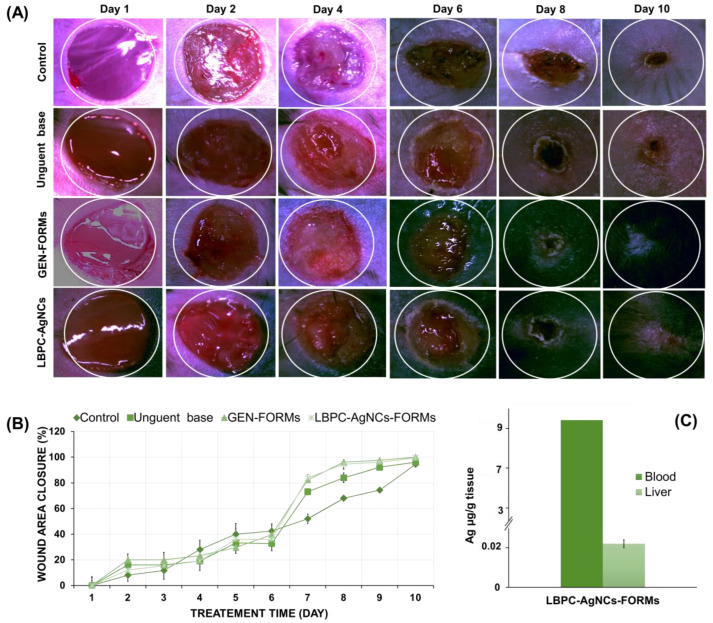
Illustration (**A**) and graphic representation (**B**) of the dynamic of wound-area closure, represented in %, based on three measurements during 10 day-monitoring and the Ag concentration in liver and blood samples (**C**).

During the recovery period, on each day the three measurements were independently taken and the results are presented as the mean ± SD. The ANOVA assay showed no statically significant differences between the first four days (*p* = 0.587). Figure 2A,B illustrates the dynamics of the wound closure process. The results show that the healing process took place in a gradual manner, presenting the regeneration effect in around 30% of mice even from the fourth day.

Mouse wounds treated with the silver formulation (LBPC-AgNCs) were closed by day 7 in around 80% of mice in contrast to the control group, whose wounds remained slightly open until day 10. In turn, for LBPC-AgNCs treatment, wound closure reached approximately 100% by 10 days. Similarly, it has been noted that, for GEN-FORMs, an antibiotic-dedicated routine is recommended for the treatment of hard-to-heal wounds. Once the wounds were totally healed during the 10 days, it was indicated that this time is sufficient for biomedical application in diabetic wound attenuation and/or for an antiseptic effect in bacterial infection.

Moreover, our previous work [14] has demonstrated the antimicrobial potential of the silver NPs investigated in the present study against various Gram (+) and Gram (−) strains. It is worth noting that, in case of wounds that were treated with the silver formulation, a rapid and dense hair growth was observed even from day 4 of the treatment compared to the control. A similar phenomenon was observed in the case of antibiotic treatment.

### 2.3. Uptake of Ag in Liver Tissue and Blood Samples

**Figure 3:**
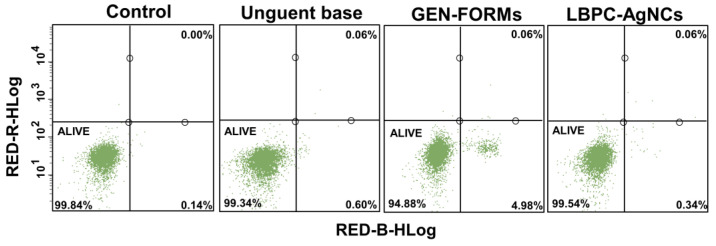
Dot–plot representation of the control, unguent base, GEN-FORMs and LBPC-AgNCs, indicating the viability of the PBMCs under external treatment.

The therapeutic effect of silver NPs is widely described and demonstrated continuously. The migration, accumulation, and speciation ability of ionic silver and AgNPs have been also demonstrated. Additionally, their cytotoxic effect on the skin, liver, brain, and blood has been reported to be dependent on the size, concentration, synthesis method, and administration route. The precise action mechanism it is still unclear, however, it has been postulated that the penetration and accumulation of the Ag+/AgNPs are the result of NP’s contact with the cell walls, followed by an uncontrolled secondary releasing of silver ions which bind to cysteine by thiol groups based on the “Trojan horse” mechanism [15]. On another hand, there is accumulation in the liver rather than distribution via the bloodstream and initiation of the uptake mechanism [6]. For these reasons, the implementation of ICP-MS in the present study is indisputable. Therefore, in the current research on therapeutic effects of LBPC-AgNCs, the accumulation potential in the liver and blood has also been estimated. Figure 3 presents the silver concentration in blood and liver samples, showing a silver concentration of <0.1 ppm (9.3 µg/g (0.0093 ppm) in blood and 0.022 µg/g in liver); this is much lower than reported in the literature when the nanomaterials were incubated for a certain period of time with the tissue (in the liver at 0.029 ppm; in blood at 0.049 ppm) [9,16]. Such observations indicate a lack of silver migration to the remote organs that consequently proves the non-toxic effect. In order to repeatedly prove the toxic effect, the viability of PBMCs was also investigated.

### 2.4. Viability of PBMC Cells–Ag Cytotoxicity Effect: Flow Cytometry Analysis

The viability assay was carried out with a Flow cytometry assay using an Annexin affinity test. The PBMC samples, collected on the last day of the experiment, were directly investigated for the detection of their apoptosis level after 10 days of external administration. The PBMCs collected from the mice treated with the LBPC-AgNCs formulation were compared with the PBMCs collected from the untreated mice (control), who were treated only with an unguent base and with GEN-FORMs.

In all cases, the viability of the cells was found to be >99%, excepting the samples of PBMCs collected from the mice that were treated with GEN-FORMs where the viable cells were equal to 94%. Very close values were found, indicating the luck of the cytotoxic effect (Figure 3). Moreover, the effect of LBPC-AgNC was compared with various nanocomposites (comparative formulations) obtained also via the biological method that could also be used as potential non-toxic agents in wound-infection treatment. In all swatches, the viability of the cells was established to be >97%, suggesting a safe effect, similar to the case of the LBPC-AgNCs formulation (Figure 3). The concentrations of the comparative formulations in present research have been chosen based on the data previously reported by our group, ZnONPs and ZnOVA (at 172.5 µg/mL) [17], whereas AgBLG and ZnBLG had concentrations of 25 µg/mL and 200 µg/mL, respectively (unpublished data). Zn nanocomposites have been investigated to exhibit both 100% of the MIC as well as 110% of the MIC (Figure S1). In the case of comparative formulations, the experiments have been conducted following the same steps as for LBPC-AgNCs, GEN-FORMs, and the control. Such formulations have been taken into consideration to have a wider spectrum of interest in the context of the delivery of silver’s effect to blood cells, and the impact of the functionalized nanoparticles (post-synthetic functionalization) on immunomodulatory effect has been reported [18]. Moreover, immunomodulatory action has been demonstrated to have importance for cancer therapy. Hence, the finding of new strategies in the context of (Bio)nanocomposites naturally functionalized with the organic deposit that will be toxic for abnormal cells and at the same time non-toxic for normal cells is of the highest interest [19]. Furthermore, due to lack of information in the literature regarding the cytotoxic effect on PBMCs of nanomaterial synthesized by biological method, which are naturally coated by organic deposits, the current investigation has compared the LBPC-AgNC’s effect with other various (Bio)nanocomposites that were biologically synthesized and characterized previously by our group.

## 3. Discussion

Recently, the complex approach (ICP/MS-microscopy/auto-metallography/histopathology) revealed focal accumulations of Ag and/or Ag-NP in sections of peripheral organs: mediastinal lymph nodes contained Ag-NP, especially in peripheral macrophages, and Ag was found in argyrophilic fibers. In the kidney, Ag had accumulated within the proximal tubule while renal filter structures contained no Ag. Discrete localizations were also observed in immune cells of the liver and spleen. Overall, the study showed that concentrations of Ag-NP, which elicit a transient inflammation in the rat lung, lead to focal accumulations of Ag in peripheral organs. This might pose a risk to particular cell populations in remote sites [10,20]. In turn, the histopathology study—another complimentary method—highlights the tissue distribution of silver (Ag) nanoparticles influenced by the dose-dependent accumulation of Ag in all the tissues examined, including testes, kidneys, liver, brain, lungs, and blood [10,21]. Additionally, a gender-related difference in the accumulation of Ag was noted in the kidneys, with a twofold higher concentration in female kidneys compared to male kidneys after subacute exposure to Ag nanoparticles via inhalation or oral ingestion [20].

Increasing attention is being given to the search for new therapies, and researchers and doctors are encouraged to search and develop feedback to solve this problem. Silver has been considered one of the earliest precious metals with remarkable healing properties. Even before antibiotic discovery, silver was found to prevent and/or treat infections [22,23,24].

The nanocomposites used have been identified as complex structures consisting of a metallic core and organic deposits; the organic deposits represent the metabolites secreted by the bacterial strain during the inoculation step [15]. Naturally-coated nanocomposites are supposed to induce controlled agent delivery systems. The present study is aimed at investigating this aspect.

The first observation noted in the present study—for instance, the decrease in moderate body weight and the atypical behavior of the mice—can be explained by the discomfort generated by the surgery, dressing process, and itching of the wound site. However, use of the silver formulation demonstrated an accelerated return to the initial body mass. During the final recovery period, no abnormal mouse behavior was observed, and there was no significant difference in body weight between treated and control mice. All mice appeared healthy.

It is important to note that, in cases of wounds that were treated with the silver formulation, a rapid and dense hair growth was observed when compared to the control, even from day 4 of the treatment. A similar phenomenon was observed in cases of antibiotic treatment. The obtained results designed the hypothesis that the LBPC-AgNCs operate as an antibiotics. It is noteworthy that the same action mechanism of the same nanocomposites investigated in the present research on the mice model has been noticed and reported by our group previously during testing against pathogenic microorganisms. This fact suggests and proves a high potential application in wound healing infection.

Many researchers have demonstrated in vitro, in vivo, and ex vivo studies relating the impact of the various nanomaterials on the viability of the prokaryotic and eukaryotic cells [24,25]. With an increase in wound infection rates, this aspect is continuously debated: on one side, the debate relates to the cytotoxicity of the nanomaterials; on the other side, the debate focuses on the therapeutic effects against malignant cells. In 2018, when Ta-Li et al. showed the toxicity of C-AgNP20 at high concentrations (10 and 50 μg/mL), their group induced a time- and dose-dependent cytotoxicity in cPMNs and cPBMCs (isolated from healthy dolphins) and an apoptotic pathway was involved in the C-AgNP20-induced cytotoxicity in vitro [26]. Additionally, significant inhibitions in the viability and proliferation process of the A2780 cell line after 24 h of incubation with AgNPs have been reported [27]. Full peripheral blood induced the production of pro-inflammatory IL−6 by monocytes [25], whereas intracellular reactive oxygen species generation, DNA damage and apoptosis (after 3, 6 and 12 h) were observed in a dose-dependent response (0.1–30 µg/mL) [28]. The negative effect of silver nanomaterial on livers was also demonstrated; the liver could accumulate silver. In turn, during long-term oral administration of silver nanoparticles it was noticed that, from the 180-day point, even with constant exposure to nanoparticles silver is washed out of the mouse liver (C57Bl/6 mice model) [29]. Additionally, an effect of fast elimination of silver from the liver and blood has been reported; one month after its withdrawal, approximately 80% of the silver was washed out. The quantification of the minimal concentration of the silver from these organs after two months of administration is explained by the large number of cells in the immune system and a relatively fast metabolism [29].

## 4. Materials and Methods

### 4.1. AgNCs Description

The AgNCs used in the present research were previously synthesized and characterized using a complimentary approach [15]. These results characterized silver NCs as complex structures consisting of a silver core naturally coated by organic deposits (metabolites secreted by bacterial strain). The synthesized AgNCs have been found to be made of nanoparticles with an average size (silver core) equal to 18 ± 2.4 nm, while the hydrodynamic size of biocolloids (nanocomposite) is about 194 nm. The size of silver nanoparticles was investigated by transmission electron microscopy technique and the hydrodynamic size value of the complex nanostructure was generated by Dynamic Light Scattering technology. Additionally, the minimal concentration that is optimal for inhibiting bacterial growth has been investigated; the obtained minimal inhibitory concentration value has been used in the present study.

### 4.2. Experimental Design: Animal Acclimatization, Body and Mice Weights, Wound Formation/Treatment and Biological Material Collection

**Figure 4:**
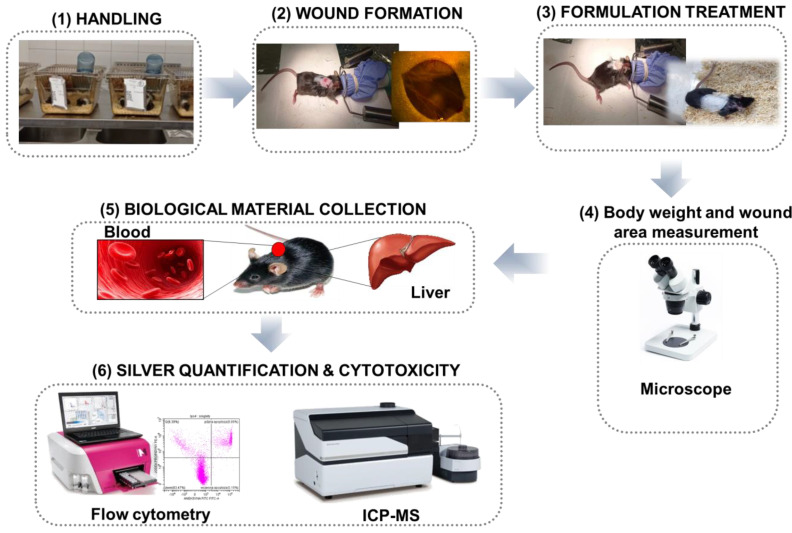
Design representation of the scheme protocol followed in the present research.

For the present study, the C57BL/6J mice aged 7–8 weeks were acquired from Tri-City Academic Animal Laboratory Research and Service Center at the Medical University of Gdansk, Poland and divided into 8 animals per group with a mean weight within the range of 21.53 ± 1.59 g. The conducted experiment has been designed in several steps (Figure 4). The first step involved handling: during this step, the mice were acclimatized for a one-week period to the controlled environmental conditions with an air temperature of 20–24 °C, humidity of 55% ± 10%, a rate of air exchange in the room of 15–20 changes/1 h; an automated light cycle with a sequence of 12 h light/12 h darkness, and free access to water and a standard pellet diet ad libitum. The second step involved wound formation: at this step, mice were first given isoflurane anesthesia (5%) via inhalation (flow rate 3.5 L/min) (Combi-Vet Anesthesia Machine Rothacher-Medical GmbH, Bern, Switzerland, after which round wounds (6 mm), were created by an excision of the skin with surgery scissors, a process that was monitored using a stereoscopic microscope with cold light (100 W–MST 132 Lab, Optika microscopes Italy, Italy). The third step involved LBPC-AgNCs formulation treatment: this stage was performed by manually dispersing the silver unguent onto the wound once per day. The formulations were prepared by incorporating the bioactive substance into a mass of cocoa butter using gentle movements based on the Minimum Inhibitory Concentration (MIC) value reported previously by our group: LBPC-AgNCs (3.12 µg/mL, final concentration) [15], Gentamicin (30 µg, final concentration) [30]. Such concentrations have been chosen based on our previous investigations where different concentrations were investigated; the concentrations used in the present research were represented by the MIC values recorded in the previous publication. The concentration has been chosen to include a wider spectrum of action. Three control groups have been taken into consideration: the first group included mice without any treatment, the second group included mice treated only with the unguent base without an active agent, and the third group included mice whose wounds were lubricated with the antibiotic formulation (Gentamicin (Sigma Aldrich, Poznań, Poland), recommended for the bedsore wound).

The lubrication wounds were covered with a sterile plaster to reduce the risk of wound infection. In the fourth step, which involved body weight and wound area measurement, body weight was measured each day and the effect of the obtained formulations on the wound closure was monitored via stereoscopic microscope combined with a cold light source (100 W–MST 132 Lab, Optika microscopes Italy, Italy). The precise wound size and volume (V) was measured for each group (8 animals per group, keeping the same conditions) and represented in photographic form. The % of wound closuring was calculated as follow: V [%] = (V _day n_/V _day 1_) ∗ 100%. A free available platform (https://goodcalculators.com/one-way-anova-calculator/ accessed on 17 October 2022) was used to show the significance changes. In the fifth step, biological material collection (blood and liver tissue) occurred. Before data collection, the rodents were euthanized 10 days after the treatment by intraperitoneal injection of sodium pentobarbital (200 mg/kg).

The collected liver tissue samples were immediately frozen and stored in −80 °C to deactivate the enzymatic reaction. The blood was collected into sodium citrate anticoagulant (3.2%) tubes. Step six involved the total silver quantification in collected samples and an analysis of LBPC-AgNC’s cytotoxicity in PBMCs (Peripheral blood mononuclear cells). The experiment took place in 10 days and was fully approved by the Local Animal Care and Use Committee in Bydgoszcz (BYD 21/2021, 26 April 2021).

### 4.3. Uptake of Ag in Liver Tissue and Blood Samples: Inductively Coupled Plasma Mass Spectrometry (ICP-MS) Analysis

The ICP-MS (Shimadzu ICP-MS 2030, Shimadzu, Kyoto, Japan) technique, equipped with a collision cell with a helium flow of 6 mL/min, was used to quantify the silver concentration (silver ion and AgNPs) in the liver tissue and blood samples. All collected biological materials were pre-digested for 4 h at 80 °C using a Thermomixer (Thermomixer comfort, Eppendorf SE, Hamburg, Germany) in 150 µL of HNO3 (1%) (Merck Suprapure, Merck, Darmstadt, Germany). Prior to analysis, the cooled samples were diluted 100 times in 1% HNO_3_. Measurements were performed on 107Ag and 103Rh as internal standards, using the method of external calibration in three replications. The results were presented in the form of a concentration unit. The concentration was reported based on the calibration curve carry-out for silver standards (Silver and Zinc Standards for ICP, Sigma-Aldrich, Merck, Germany) in a concentration range of 0—25 µg/L.

### 4.4. Viability of Peripheral Blood Mononuclear Cells—Ag Cytotoxicity Effect: Flow Cytometry Analysis

The Flow cytometry assay was divided in two parts: (i) the isolation of PBMCs and (ii) the cytotoxicity effect investigation. Step one was carried out by diluting the blood samples with sterile phosphate-buffered saline (PBS) in equal volume and slowly layering them onto the lymphocyte separation medium. Then, after centrifugation (500× *g*, 30 min at 20 °C), a thin layer of mononuclear cells was gently transferred to a new sterile tube and washed with PBS three times. The obtained cells were resuspended in RPMI−1640 medium (supplemented with 10% fetal bovine serum, 2 mM L-glutamine, 50 U mL^−1^ penicillin, and 50 mg mL^−1^ streptomycin). Afterwards, the cells were counted (in triplicate) using a Trypan blue exclusion assay and a hemocytometer with a light microscope (LABOMED microscope CXL, Fremont, using a hemocytometer in Fremont, CA, USA). Once the PBMCs were isolated, they were adjusted to 2.5 × 105, divided into 96-well plates (Corning Incorporated, Corning, NY, USA) and subjected to the cytotoxicity assay (Step (ii)). In the second step, freshly prepared plates were processed according to the FlowCellectTM Annexin Red Kit guidelines recommended by the manufacturer in order to identify the silver cytotoxic potential. All reagents used for the flow cytometry analysis were purchased from the Merck Life Science Sp. z o.o., Poznań, Poland. Prior to each experiment, the technique was set according to the Flow Cytometer Guava EasyCyte 8HT (Merck Millipore Corporation, Warsaw, Poland) system Flow cell instruction. The performance of the easyCyte system was conducted according to the Guava ICF instrument cleaning guidelines (Merck Millipore Corporation, Warsaw, Poland).

## 5. Conclusions

In the present study, after external administration of LBPC-AgNCs (for 10 days), the cells’ viability was estimated to be very high (>97%), though insignificant traces of silver were registered in the blood and liver. The tight relationship between these two parameters could be explained by the control delivery system exercised by the organic matrix present in the nanocomposites’ structure.

In contrast to the literature data, the present report becomes a pilot study in transferring the in vitro study to in vivo scale (e.g., medical field application) once LBPC-AgNCs have demonstrated a unique wound-healing potential as well as a non-toxic effect. Moreover, the present survey supports the previous hypothesis and emphasizes once again the use of naturally functionalized nanocomposites against various skin infections and highlights the impact of organic deposits.

## Data Availability

Not applicable.

## Supplementary Materials

The following supporting information can be downloaded at: https://www.mdpi.com/article/10.3390/ijms24010434/s1.
