# Supplementary material for: In Vivo Efficacy of Wound Healing under External (Bio)AgNCs Treatment: Localization Case Study in Liver and Blood Tissue
Source: Int J Mol Sci. 2022 Dec 27;24(1):434. doi: 10.3390/ijms24010434 (PMC9820314; doi:10.3390/ijms24010434)
Supplement: Supplementary file 1 [file ijms-24-00434-s001.zip › ijms-2008025-supplementary.pdf]

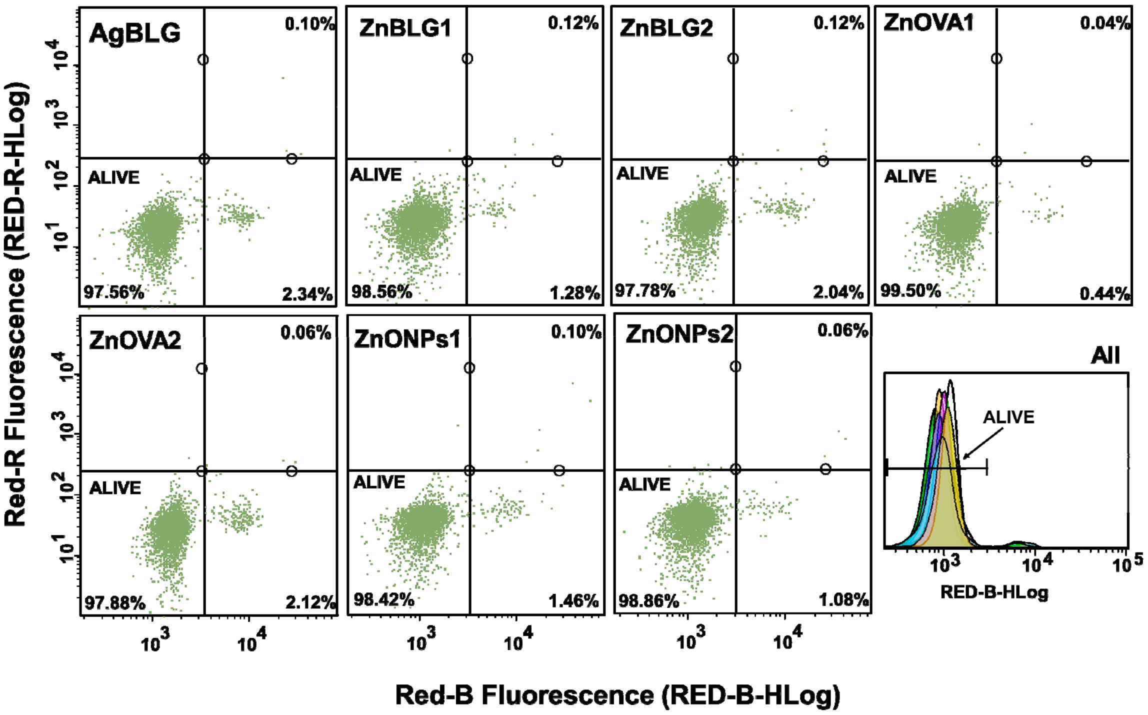

Figure S1. Dot plot representation of different nanocomposites synthesized by biological method that.
